# A Novel Framework for Quantifying Accuracy and Precision of Event Detection Algorithms in FES-Cycling

**DOI:** 10.3390/s21134571

**Published:** 2021-07-03

**Authors:** Ronan Le Guillou, Martin Schmoll, Benoît Sijobert, David Lobato Borges, Emerson Fachin-Martins, Henrique Resende, Roger Pissard-Gibollet, Charles Fattal, Christine Azevedo Coste

**Affiliations:** 1 National Institute for Research in Computer Science and Automation (Inria), Camin Team, 34090 Montpellier, France; sijobert.b@institut-st-pierre.fr (B.S.); christine.azevedo@inria.fr (C.A.C.); 2 Le Laboratoire d’Informatique, de Robotique et de Microélectronique de Montpellier (LIRMM), Université de Montpellier (UM), 34090 Montpellier, France; martin.schmoll@meduniwien.ac.at; 3 Institut Saint-Pierre (ISP), 34250 Palavas-les-Flots, France; 4 Núcleo de Tecnologia Assistiva, Acessibilidade e Inovação (NTAAI), Universidade de Brasília (UnB), Brasília 70910-900, Brazil; borges.david@aluno.unb.br (D.L.B.); efmartins@unb.br (E.F.-M.); 5 Departamento de Engenharia Elétrica, Universidade Federal de Minas Gerais (UFMG), Belo Horizonte 31270-901, Brazil; henriquerm@ufmg.br; 6 National Institute for Research in Computer Science and Automation (Inria), SED Service, 38330 Montbonnot, France; roger.pissard@inria.fr; 7 Rehabilitation Center Bouffard Vercelli, USSAP, 66000 Perpignan, France; cfattal@ussap.fr

**Keywords:** event detection, IMU, cyclic motion, rehabilitation, functional electrical stimulation, FES-cycling, gait cycle index

## Abstract

Functional electrical stimulation (FES) is a technique used in rehabilitation, allowing the recreation or facilitation of a movement or function, by electrically inducing the activation of targeted muscles. FES during cycling often uses activation patterns which are based on the crank angle of the pedals. Dynamic changes in their underlying predefined geometrical models (e.g., change in seating position) can lead to desynchronised contractions. Adaptive algorithms with a real-time interpretation of anatomical segments can avoid this and open new possibilities for the automatic design of stimulation patterns. However, their ability to accurately and precisely detect stimulation triggering events has to be evaluated in order to ensure their adaptability to real-case applications in various conditions. In this study, three algorithms (Hilbert, BSgonio, and Gait Cycle Index (GCI) Observer) were evaluated on passive cycling inertial data of six participants with spinal cord injury (SCI). For standardised comparison, a linear phase reference baseline was used to define target events (i.e., 10%, 40%, 60%, and 90% of the cycle’s progress). Limits of agreement (LoA) of ±10% of the cycle’s duration and Lin’s concordance correlation coefficient (CCC) were used to evaluate the accuracy and precision of the algorithm’s event detections. The delays in the detection were determined for each algorithm over 780 events. Analysis showed that the Hilbert and BSgonio algorithms validated the selected criteria (LoA: +5.17/−6.34% and +2.25/−2.51%, respectively), while the GCI Observer did not (LoA: +8.59/−27.89%). When evaluating control algorithms, it is paramount to define appropriate criteria in the context of the targeted practical application. To this end, normalising delays in event detection to the cycle’s duration enables the use of a criterion that stays invariable to changes in cadence. Lin’s CCC, comparing both linear correlation and strength of agreement between methods, also provides a reliable way of confirming comparisons between new control methods and an existing reference.

## 1. Introduction

Functional electrical stimulation (FES) is a rehabilitation technique based on the activation of muscle contractions in paralysed limbs through the application of short electrical pulses in order to recreate a function or motion [1]. In individuals with spinal cord injury (SCI), the use of FES for clinical applications activating the lower limbs has proven to increase muscle mass [2,3], to decrease risks related to prolonged sitting position such as pressure sores [4,5], and to decrease risks of cardiovascular diseases [5]. Exercises such as FES-cycling in SCI have also shown to have physiological benefits, notably on the cardiorespiratory system [6,7].

FES-cycling consists of sequentially electrically stimulating the paralysed lower limb muscles to induce a pedalling motion. Classically left and right legs hip and/or knee extensor and flexor muscles are stimulated alternatively based on the information delivered by a crank angle sensor [8,9,10]. In this context, a stimulation pattern that correlates the measured crank angle to the individual muscles’ activation is usually preset and adjusted empirically in a time-consuming process [11]. Furthermore, several variables are still subject to change throughout a cycling session, for instance, the time delay in muscular force production, which can be affected by muscle fatigue [12]. Synchronising muscle-stimulation-triggering events in relation to the crank angle presumes a fixed geometrical model dependent on the pilot’s anatomy and seating position [13]. Variations in the pilot’s seating position or in the positioning of the pilot’s legs can result in changes in the geometrical model, thus causing a misalignment of the original pattern. This can in turn decrease the efficiency of the torque production or the quality of synchronisation of the stimulation pattern [14]. Adjustments to the stimulation pattern might become necessary in such cases during use or after each installation on the bike for the system to maintain efficient timings of muscle contraction.

To address this, an approach that does not rely on a predefined geometrical model of the legs is required in order to automatically adapt stimulation timings to dynamic changes (e.g., seating position). In the last years, some research groups have proposed to replace the crank angle encoder with inertial measurement units (IMU) located on the user’s limbs [15,16,17,18]. Inertial measurement units placed on anatomical segments allow for generic and adaptive ways of defining the stimulation patterns based on leg movement criteria (i.e., maximum knee flexion/extension) [17]. While the knee angle can be determined via a set of two independent IMUs per leg located on the thigh and shin [18], it is also possible to use a more simplistic method to control stimulation during FES-cycling by measuring only the thigh angle [17]. Both approaches open new possibilities for the automatic design of stimulation patterns. In exchange for not relying on stimulation timings set for a statically predefined geometrical model, certain anatomical angles have to be interpreted in real time to follow the cycling motion and determine when to deliver stimulation. Depending on the application and its requirements, predefined limits of agreement [19] between the method to be evaluated, and the reference can be used to assess if its precision and accuracy are to be considered sufficient [20,21].

In this article, we aim to analyse the precision and accuracy of three algorithms by comparing their movement event detection results with the desired targets (i.e., relative time points within a cycle at which the onset of stimulation is to be triggered) occurring on a post-calculated reference baseline. In contrast to techniques which track the knee-angle [18] or control stimulation based on the crank angle [10,22,23,24], we decided to evaluate algorithms which can directly interpret the thigh inclination (measured by a single IMU) as the common input signal. The selected algorithms allowed us to detect the desired stimulation-triggering events throughout consecutive cycles. The first algorithm, called BSgonio, was described in a previous article [17]. The second one, called Gait Cycle Index (GCI) Observer, was adapted from a cyclic motion phase estimator [25] based on an oscillator mimicking the behaviour of a central pattern generator (CPG) [26] and tested in gait monitoring [25,27]. The third one, called Hilbert, is based on the instantaneous phase function of the Hilbert transform, a posteriori generated, which interprets rhythmic signals as a cyclic phase [28,29]. We hypothesised that the algorithms will perform accurate and precise event detection, compared to a post-calculated baseline, within predefined limits of agreement of ±10% of the cycle’s duration. Further, we present a method for quantifying the deviations from the targeted baseline events to compare the accuracy and precision of the individual algorithms. 

## 2. Materials and Methods

### 2.1. Subjects

Six participants with complete SCI (Age: 39 ± 5 years) were recruited for this study after signing a written informed consent. They were participating in an FES training protocol which was approved by the Ethics and Research Committee of the Science Health Faculty, University of Brasília, Brasília, Brazil (CAAE: 50337215.1.0000.0030, Ethical Approval number 1.413.934). Detailed information on the participants can be found in Table 1. The inclusion criteria were (i) complete spinal cord injury, (ii) single neurological level between C5 and T12, (iii) time interval since SCI > 12 months (with a stable ASIA motor score > 6 months), (iv) unrestricted joint movement and (v) body mass index < 30. Exclusion criteria were (i) epilepsy, (ii) cardiovascular disease, and (iii) fractures of the lower limbs within the last 12 months.

### 2.2. Material

**Figure 1:**
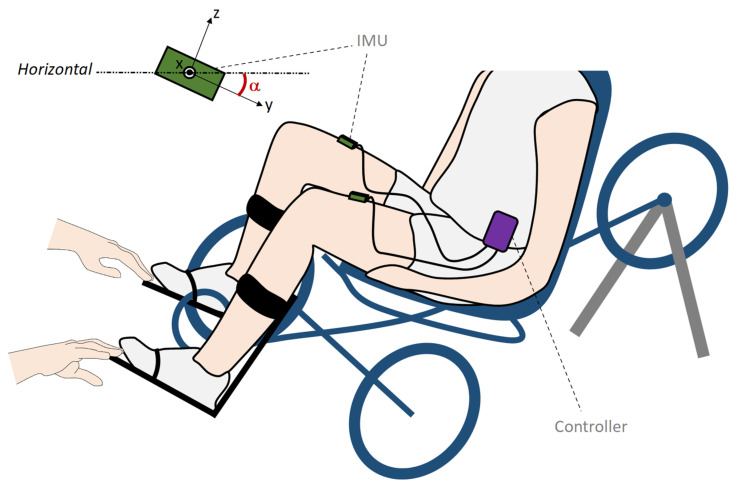
Experimental setup for cycling movement analysis. Participants were equipped with the IMUSEF system composed of two inertial measurement units, one located on each thigh and connected to a controller (Raspberry Pi 3B© embedded single board computer) used to acquire the inertial measurement data. The IMU’s axes are represented in the top left with the thigh angle inclination being the α angle (gyroscope roll axis).

Data acquisition was made with the participants positioned in a recumbent tricycle (Trike Full Suspension, Arttrike, Brazil) mounted on a home trainer (IN RIDE 100 B'TWIN, Decathlon, France) (Figure 1). Orthotic calf supports (Hase special pedals v1, HaseBikes, Germany), fixed to the pedals, were used to securely strap the participants’ feet and restrain ankle motion. Each leg was equipped with an inertial measurement unit (IMU, Bosch© BNO055, Gerlingen, Germany) wired to a processing unit (Raspberry Pi 3B ©, Raspberry Pi Foundation, Cambridge, UK) running a Python/C++ program for online data acquisition and motion analysis called IMUSEF (CAMIN, INRIA, Montpellier, France), which implemented the tested online algorithms. Each IMU embedded a high-speed ARM Cortex-M0-based processor and a Kalman filter directly able to provide the quaternion calculation needed to compute thigh inclination angles in reference to the horizontal plane (i.e., flat ground). The inertial data were calculated by the IMU modules at a sampling rate of 100 Hz and were pulled by the controller through a wired I2C communication interface. A posteriori data processing and statistical analysis were performed in MATLAB 2016a (MathWorks, Natick, MA, USA).

### 2.3. Methods

#### 2.3.1. Data Acquisition and Conditioning

The inertial data were recorded while the legs of the subjects were passively moved by an experimenter. The average sampling frequency of the recording system was 63 Hz. Although this sampling frequency was high enough for real-time data control, it was too low for the precise evaluation of motion analysis algorithms. In fact, the average sampling period being 16 ms, when cycling, for example, at a cadence of 60 revolutions per minute (RPM), it amounts to 1.6% in cycle duration between each sample. To compensate for this, we upsampled the input data a posteriori using MATLAB’s linear interpolation function (interp1) to increase the input thigh angle inclination sampling frequency to 1000 Hz. Using this frequency, the sampling imprecision from the previous example was reduced to 0.1%. The 1000 Hz frequency was chosen because it was the maximum sampling frequency that the GCI algorithm was able to use for its input model.

#### 2.3.2. Baseline Reference for Event Detection

To evaluate each algorithm’s accuracy and precision, their detected events had to be compared to the targeted events. A baseline reference containing the targeted event detections was produced by computing the cyclic phase of the thigh angle input signal. This phase represents the progression of a cycle in percentage. Cycles were delimited using the peaks from the thigh angle cyclic movement, from a peak of leg extension to the next one.

**Figure 2:**
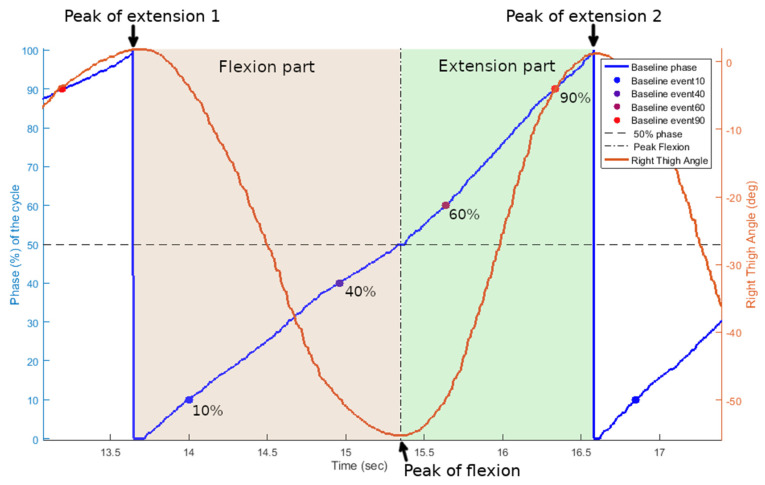
Data sample from subject 5. Progression of the cycling movement as a linear phase increasing throughout a pedalling cycle and used as the baseline for reference in comparisons. Inclination angle of the right thigh in degrees during the cycling movement is shown in red (right axis). Phase in percentage of the cycle’s progress is shown in blue (left axis). Events for threshold levels (10%, 40%, 60%, and 90%) from the baseline phase are shown in a gradient of coloured dots. Flexion and extension phases of the pedalling cycle are highlighted, respectively, with light red and light green areas.

The cyclic phase of the baseline is a linearised normalisation of the thigh angle signal calculated for each cycle individually. The cycles were first divided into two parts, flexion (0 to 50% of the cycle) and extension (50 to 100% of the cycle), as can be seen in Figure 2. The first part, constituting the flexion half of the cycle, is delimited by the starting peak (local maxima), indicating a fully extended leg, and the following peak (local minima), indicating the completed leg flexion. Normalisation is applied using the range of motion in angle values between those two peaks. The second part, continuing from the same peak of flexion until the next peak of extension, marking the end of the current cycle, is again normalised by the angle values of its two peaks. These two normalised parts are then linearised through the arc cosine function as the input signal is originating from a circular motion. This linearised normalised phase is then finally scaled to fit percentile values from 0 to 100.

The performances of the algorithms were evaluated for thresholds ±10% around peak flexion and peak extension of the leg to represent key events in each pedalling cycle. The chosen events were therefore defined for each cycle in the baseline as the threshold values 10%, 40%, 60%, and 90%.

#### 2.3.3. Algorithms 

Three algorithms were evaluated to detect recurrent events throughout the pedalling cycles. All of them were using the thigh angle (i.e., angle of the thigh relative to the horizontal plane) as input. The ones called BSgonio and GCI Observer were generating their outputs in IMUSEF (custom-developed data acquisition and motion analysis software). The third one, called Hilbert, intended as a control method for our standard of comparison, was computed a posteriori in MATLAB.

BSgonio

The algorithm called BSgonio, described in a previous article [17], is based on online peak detection of the motion of a joint or anatomical segment. After each new peak detection, thresholds relative to the angle value of the corresponding peak are updated. These adaptive thresholds are the latest value of the corresponding peak, minus an offset allowing the definition of the desired event detection. This offset can be empirically adapted to correspond to the desired event. For this study, the range of motion was preset to a constant value of 40 degrees (average of all subjects). The general equation that determines the desired threshold in this approach for a given phase value can be represented as follows:

(1) Th=Lpk_ext−(ROM−(cos(2π×x)+1)×ROM/2)

where x is the desired detection phase in percentage, ROM is the anticipated or last known range of motion, Lpk_ext is the last peak of extension value in degrees, and Th is the resulting angle threshold for the event detection in degrees. In this case, for an offset of 10% around a peak (flexion or extension), when the input angle has a range of motion of around 40 degrees, an internal offset value of 3.82 degrees can be used to detect the desired thresholds within this study (i.e., 10%, 40%, 60% and 90% of the baseline).

GCI Observer

The GCI Observer algorithm uses a motion analysis model based on a nonlinear oscillator that mimics the natural central pattern generator’s control strategies [26]. This motion analysis algorithm was described and used in hemiplegic gait monitoring and ankle joint control with the use of FES to assist with the drop-foot syndrome in hemiplegic subjects [27]. By analysing a cyclic signal and comparing it to a reference model, it is able to generate in real time an estimation of the progression of a rhythmic motion.

The reference model that was used for all subjects was a sine curve representing the thigh angle during a cycling motion. The GCI algorithm compares the measured thigh angle at its corresponding angular velocity with the reference model to infer the progress of the current cycle. This progress output is given as a linear phase in percentage.

Hilbert

The third algorithm, called Hilbert, is based on the Hilbert transform, a signal processing method with multiple properties [29]. The one used here is the instantaneous-phase function which results from the complex representation of the input signal obtained through this transformation. The Hilbert transform outputs an analytical representation of the input signal as complex numbers with real and imaginary parts. Observing the phase angle of the resulting complex function yields the phase representation for a periodic signal. The last step is to normalise this phase representation to percentile values from 0 to 100. The complete process in MATLAB is as follows:

(2) output_phase=((angle(hilbert(input_signal))/π)+1)×50

Although the calculation was carried out a posteriori, we consider the widely used Hilbert function a valuable algorithm for comparison. However, this method cannot be efficiently used in real time as discontinuities in the input signal are not accurately processed by the Hilbert transform.

#### 2.3.4. Event Detection

For the BSgonio method, the events were detected by scanning through the thigh angle signal and comparing it to the four predefined offset values (i.e., offsets corresponding to the threshold values of the baseline) relative to the latest peaks. When the thigh angle value passed a given threshold, the corresponding event was detected. For the GCI and Hilbert algorithms, due to the output value being already a phase representation in percentage, their events were directly detected when the phase value passed the predefined target thresholds. Next, each event detected by an algorithm was paired with its corresponding target event of the baseline reference. The delay between the two was computed in percentage of the cycle’s duration.

Whenever an algorithm had false-positive event detections (e.g., wrong peak detection from perturbations in the cycling motion) due to a flawed behavioural interpretation, the detected events were kept to penalise the algorithm. However, false negatives (i.e., missed detections) could not be evaluated due to the unavailability of the event detection in question and hence could not be reflected in the results.

#### 2.3.5. Statistical Data Analysis

The criterion we selected for our accepted error margin was ±10% of the cycle’s duration. It corresponds to 100 ms of delay for a cadence of 60 RPM which can be considered as a reasonable upper limit for the cadence in FES-cycling with SCI subjects. Each algorithm’s event detection has to fit this criterion by having at least 95% of the detected event delays being inside of the accepted error margin. For this, the limits of agreement between each algorithm and the baseline reference are computed and displayed using Bland–Altman graphs [30]. If the LoA of an algorithm exceeds either the lower (event detected too early) or the upper (event detected too late) limits, the corresponding algorithm’s event detection is considered insufficient for practical use.

To check for the normal distribution of the results, a Shapiro–Wilk test with a significance level of α = 0.05 was applied to the delays between the detected events and the corresponding baseline’s target events. As it was not possible to verify normality for each algorithm, the non-parametric version of the Bland–Altman method was used to determine the limits of agreement. This method consists of determining the upper and lower LoA by using the 2.5 and 97.5 percentiles of the distribution.

Finally, a correlation coefficient was computed for each algorithm to confirm the strength of agreement between the algorithm’s event detections and the target references. The Lin concordance correlation coefficient (Lin’s CCC) was used as it is appropriate for evaluating the strength of agreement between two methods [31]. Lin’s CCC is a measure of agreement and correlation between raters often used in clinical settings to validate potential new measurement methods against a current gold standard [32]. Since it is a product of the Pearson correlation coefficient and the squared difference between the measurements, a high coefficient means both strong agreement and linear relationship between the methods. The interpretation of its result is performed following the McBride proposal [33].

## 3. Results

**Figure 3:**
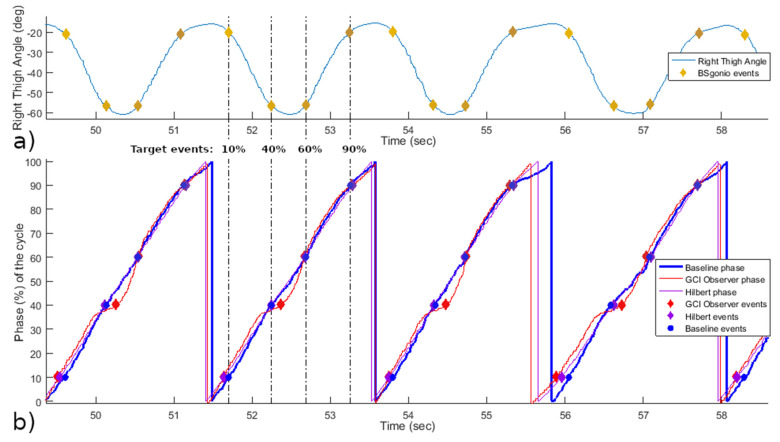
Representative data from subject 1 showing the right thigh angle on the top (**a**) and the phase estimations on the bottom (**b**). On the bottom graph, the blue line is the baseline reference, the red line is the GCI Observer output phase interpretation, and finally in purple, the Hilbert phase estimation. Corresponding events detected by the algorithms are shown superimposed on their respective line. Target events from the baseline in the second cycle are highlighted with vertical lines to help visual comparison with the corresponding event detections from the other algorithms.

The results are presented for the 6 participants combined which amounted to 195 cycles and a total of 2327 events detected between the 3 algorithms. The Hilbert algorithm had five false positives, of which one could not be associated with a target event and had to be excluded. The BSgonio algorithm had six false positives and four false negatives, while GCI Observer had none. All of these erroneous detections were due to extreme and rapid variations in the shape of the input thigh angle due to difficulties from the experimental conditions. The average cycle duration was of 2.62 seconds (i.e., cadence: 24.1 RPM) with a standard deviation of 0.64 seconds (i.e., SD of cadence: ±4.93 RPM). A representative sample of the results is shown in Figure 3. 

The Shapiro–Wilk test for normal distribution revealed that the data of all three algorithms deviated significantly from a normal distribution (*p* < 0.05). Medians, means, standard deviations, non-parametric 95% LoA, and absolute 95% LoA of the delays in event detection are shown in Table 2 for each algorithm. All values are in percentage of the cycle’s duration that the target event was part of.

**Figure 4:**
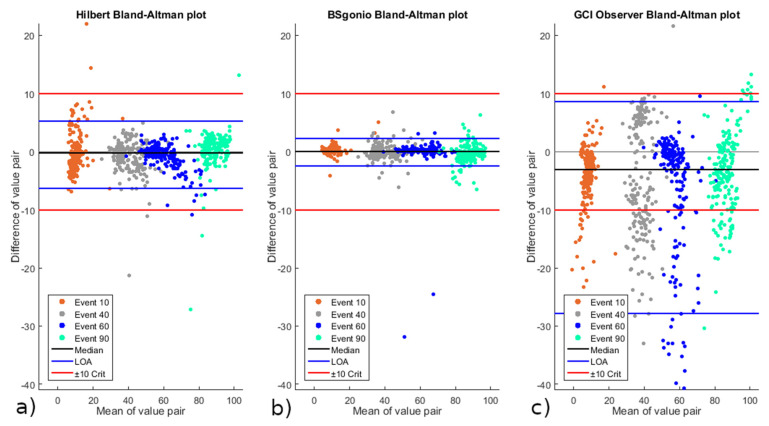
Non-parametric Bland–Altman results for the three algorithms. Presented from left to right, respectively: (**a**) Hilbert, (**b**) BSgonio, and (**c**) GCI. Event types (10, 40, 60, and 90%) are shown separately with dedicated colours. Upper and lower limits of agreement shown as blue lines; upper and lower accepted error margin criterion shown as red lines (±10%); median shown as black line; zero shown as a light grey line.

The algorithm’s limits of agreement and accepted margin of error criterion are shown in Bland–Altman plots in Figure 4, as well as the distribution of event detection results compared to the targeted reference events. Considering the selected criterion of ±10% range for accepted error, the Hilbert and BSgonio algorithms validated the criterion, while the GCI Observer did not.

**Figure 5:**
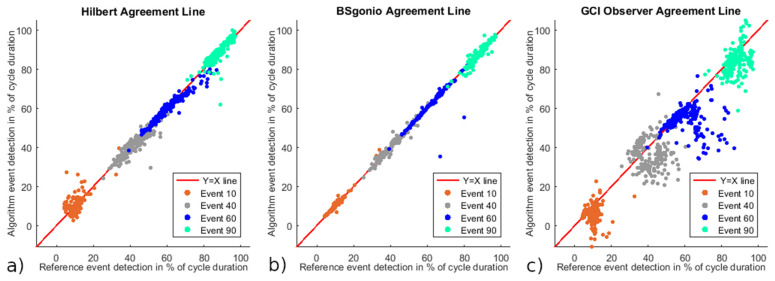
Reference event detections against Algorithm event detections for the three algorithms. The red line is the 45° line through the origin where Y = X corresponding to perfect agreement between the two methods. Presented from left to right: (**a**) Hilbert, (**b**) BSgonio, and (**c**) GCI Observer. Respective event types (10, 40, 60, and 90%) are shown separately with dedicated colours.

As part of Lin’s concordance correlation coefficient, the data are presented in Figure 5 along a 45° line of equality between the algorithm’s event detections and reference target events which corresponds to perfect agreement. Lin’s CCC and its confidence intervals (CI) are shown in Table 3 for each algorithm. Following McBride’s interpretation of Lin’s CCC, the lower CI bounds of the Hilbert and BSgonio algorithms being superior to 0.99, they can be considered as having an almost perfect agreement with the baseline reference. While the lower CI bound of the GCI Observer algorithm was comprised in the 0.90 to 0.95 range which indicates a moderate strength of agreement.

## 4. Discussion

While the use of crank angle encoders to control functional electrical stimulation had been present in literature for decades [8,9], new approaches helping to overcome some of the difficulties encountered with those systems are appearing [17]. Such adaptable algorithms allow for compensation of changes in the underlying geometrical model (e.g., due to alterations in the pilots seating position) in real time, thus ultimately could lead to an optimised timing for the delivery of electrical stimulation.

However, ensuring the reliability of such an algorithm is of uppermost importance. Even slight deviations from the well synchronised timed contractions can cause unproductive torque generation or even contractions against the desired movement. The purpose of this study was to present a methodology for evaluating the accuracy and precision of such control algorithms for FES-cycling which detects specific events within a pedalling cycle. The criterion selected here to evaluate the considered algorithm’s event detection’s reliability was an accepted margin of error of ±10% of the cycle’s duration with a 95% confidence interval requirement. Normalising delays in detection by the duration of the cycle in question allows for the criterion to ensure comparability throughout different cycles and sessions. A value of ±10% for the criterion was considered strict enough, compared to the variations of other existing delays in neuromuscular electrical stimulation (NMES) [12,13,34,35]. Rinaldin et al. (2020) [12] showed that muscular fatigue increased significantly the delay from onset of muscle stimulation to muscular force production in SCI participants from averages of 146.50 ms pre-fatigue to 199.0 ms post-fatigue. Sinclair et al. (2004) [35] also showed that both rise time and rise delay in muscle force production change depending on the angle of the knee at which the contraction is produced, averages ranging from 31 ± 3 ms to 67 ± 24 ms for rising delay and 72 ± 14 ms to 140 ± 62 ms for rising time, taking into account inter-subject variability. Since all these variations are combined and subject to change during the course of a cycling session, offset delay compensations become difficult. In order to represent this range of expected in-session variations, we chose the limit of 100 ms to be fulfilled by the algorithms. To keep the proportions of this criterion despite changes in cadence, its normalised correspondence at the fastest expected FES-cycling cadence of 60 RPM, 10% of the cycle’s duration was used.

In order to evaluate the three described algorithms, a reference was needed to quantify their respective event detection behaviours. This baseline reference was computed a posteriori to obtain reliable event detections throughout the recorded data, representing selected times in each cycle for starting and stopping the stimulation. It was calculated by taking into account the sinusoidal shape of the thigh angle during a cyclic motion and outputs the linear progression throughout the cycle in percentage. This allowed for accurate analysis and representation of the expected linear phase for each recorded cycle.

The main characteristics to take into account when evaluating timings of an algorithm are precision (or spread, represented by the standard deviation) and accuracy (or bias, represented by the mean) of the relative delays. Although in theory a static bias can be compensated, it remains an important characteristic of an algorithm. In praxis, bias compensation in real time can be difficult to achieve with some algorithms such as the GCI Observer, since its interpretation is related to the shape of the input signal and thus would require separate compensation individually for each subject, hence making bias compensation difficult or even impractical in this case. For BSgonio, bias can be compensated when using the actual range of motion instead of a predefined one. This would ensure correct offset computation even if a change in range of motion happens during use. This modification as well as a normalised cycling phase output would constitute valuable improvements to the BSgonio algorithm for future use in real-case applications. As for the last one, the Hilbert algorithm is not subject to real-time bias compensation since it is not computed in real time but rather offline with a priori knowledge of the entire signal. 

The non-parametric Bland–Altman method was used to determine the 95% limits of agreement. These LoA revealed that only the event detection of the Hilbert (−6.34% to +5.17%) and BSgonio (−2.51% to +2.25%) algorithms were inside of the defined accepted margin of error of ±10%. Their average delay errors (bias) were very small as well, respectively, −0.40% for Hilbert and −0.03% for BSgonio. In our average cycling cadence of 24.1 RPM, this resulted in an average temporal delay of −10 ms for Hilbert and −0.7 ms for BSgonio. On the other hand, GCI had a noticeably higher bias of −4.63% (i.e., −115.2 ms at 24.1 RPM). Despite an upper LoA of +8.59% (which was inside of the accepted range), its lower LoA of −27.89% did not fit the accepted margin. 

Similarly, the Lin concordance correlation coefficient, interpreted with the McBride strength-of-agreement proposal [33], deemed the GCI Observer algorithm to only have a “moderate” strength-of-agreement with the baseline reference, with a worst-case Lin’s CCC of 0.9360. Indeed, McBride proposed that the lower one-sided 95% confidence limit should be the value compared to assess the degree of agreement. Finally, the Lin CCC confirmed an “almost perfect” degree of agreement for the Hilbert and BSgonio algorithms, compared to the baseline reference, with lower confidence interval limits of 0.9932 and 0.9966, respectively.

Limitations of this study were the small number of SCI subjects and that the inertial measurement data were acquired from the passive movement of the legs entertained by manual rotation of the pedals from the experimental investigators rather than from autonomous FES-induced cycling. Therefore, the presented results are to be interpreted with care. However, as demonstrated in Sijobert et al. [17], the BSgonio algorithm has already proven to be able to control electrical stimulation accurately and precisely, allowing for FES-induced cycling. Further, while the recorded cycling motion might differ from FES-induced pedalling, we are confident that the variability of the movement that was introduced by the experimental investigator is sufficient for emulating the general characteristics and boundaries of FES-cycling. Regarding the small number of subjects, it is important to note that a total of 780 events were presented to each algorithm which we consider to be sufficient for a quantitative assessment of accuracy and precision.

Having algorithms that are adaptive and reliable for the real-time interpretation of anatomical segments of subjects can facilitate the use of FES systems in practical applications. However, the accuracy and precision of these real-time interpretation algorithms need to be evaluated to allow for an objective comparison. With this article, we present a novel methodology for evaluating and quantifying the accuracy and precision of real-time control algorithms for FES-cycling. The threshold-based event detections of three algorithms (BSgonio, GCI, and Hilbert) were analysed during 195 passive pedalling cycles of inertial measurement data obtained from 6 participants with SCI. An algorithm was deemed usable if at least 95% of all its detected events fitted within a predefined accepted margin of error of ±10% of the cycle’s duration. Based on our analysis, the BSgonio algorithm proved an accurate and precise way of detecting events reliably in real time. The GCI Observer, on the other hand, failed our evaluation criteria and might therefore be insufficient during practical use in FES-cycling. The Hilbert algorithm was used as a control method to confirm the validity of our evaluation process and showed only minor deviations to the baseline reference. In addition to the evaluation of the presented algorithms for FES-cycling, our method of comparison could be easily modified to be relevant in other fields of application, where algorithmic accuracy and precision need to be assessed.

## Figures and Tables

**Table 1:** Participants’ specific inclusion data.

Subject	Gender	Age (Years)	Body Mass Index (kg/m^2^)	Time Since Injury (Months)	Injury Level
P1	Male	43	19.4	252	C5
P2	Male	33	23.4	204	C5
P3	Male	42	29.4	36	T6
P4	Male	33	29.2	144	T1
P5	Male	47	24.0	79	T7
P6	Female	36	21.4	180	T7
Mean	-	39	24.5	149	-
SD ^1^	-	5	3.7	73	-

^1^ SD: standard deviation.

**Table 2:** Algorithms respective results presented in percentage of cycle duration.

	Hilbert (n = 784)	BSgonio (n = 770)	GCI (n = 772)
	Delay in % of cycle duration	Delay in % of cycle duration	Delay in % of cycle duration
Median delay error	−0.20	0.05	−3.08
Mean delay error ± SD	−0.40 ± 3.16	−0.03 ± 2.22	−4.63 ± 8.70
95% Upper LoA	5.17	2.25	8.59
95% Lower LoA	−6.34	−2.51	−27.89
Absolute 95% LoA	5.59	2.44	21.57

**Table 3:** Algorithms respective Lin’s concordance correlation coefficients and confidence intervals.

	Hilbert	BSgonio	GCI
Lin CCC	0.9941	0.9971	0.9440
Upper 95% of CI	0.9948	0.9975	0.9511
Lower 95% of CI	0.9932	0.9966	0.9360

## Data Availability

Data sharing is not applicable to this article.
